# Differences in Efficacy and Safety of Pharmaceutical Treatments between Men and Women: An Umbrella Review

**DOI:** 10.1371/journal.pone.0011895

**Published:** 2010-07-30

**Authors:** Gerald Gartlehner, Andrea Chapman, Michaela Strobelberger, Kylie Thaler

**Affiliations:** 1 Department for Evidence-Based Medicine and Clinical Epidemiology, Danube University Krems, Krems, Austria; 2 Sheps Center for Health Services Research, University of North Carolina at Chapel Hill, Chapel Hill, North Carolina, United States of America; Universidad Peruana Cayetano Heredia, Peru

## Abstract

Being male or female is an important determinant of risks for certain diseases, patterns of illness and life expectancy. Although differences in risks for and prognoses of several diseases have been well documented, sex-based differences in responses to pharmaceutical treatments and accompanying risks of adverse events are less clear. The objective of this umbrella review was to determine whether clinically relevant differences in efficacy and safety of commonly prescribed medications exist between men and women. We retrieved all available systematic reviews of the Oregon Drug Effectiveness Review Project published before January 2010. Two persons independently reviewed each report to identify relevant studies. We dually abstracted data from the original publications into standardized forms. We synthesized the available evidence for each drug class and rated its quality applying the GRADE (Grading of Recommendations Assessment, Development and Evaluation) approach. Findings, based on 59 studies and data of more than 250,000 patients suggested that for the majority of drugs no substantial differences in efficacy and safety exist between men and women. Some clinically important exceptions, however, were apparent: women experienced substantially lower response rates with newer antiemetics than men (45% vs. 58%; relative risk 1.49, 95% confidence interval 1.35–1.64); men had higher rates of sexual dysfunction than women while on paroxetine for major depressive disorder; women discontinued lovastatin more frequently than men because of adverse events. Overall, for the majority of drugs sex does not appear to be a factor that has to be taken into consideration when choosing a drug treatment. The available body of evidence, however, was limited in quality and quantity, confining the range and certainty of our conclusions.

## Introduction

Being male or female is an important determinant of societal roles, individual and health behaviors, risks for certain diseases, as well as patterns of illness and life expectancy. In recent years, the importance of potential differences between men and women has resulted in considerable effort to understand the role of sex in health and disease [1], [2], [3].

In 2001 the United States(U.S.) Institute of Medicine released a report that confirmed differences between men and women in the prevalence and severity of a broad range of diseases and conditions [3]. For example, depression, irritable bowel syndrome, urinary incontinence, or osteoporosis affect women more commonly than men, while coronary artery disease, autism, and learning disabilities occur more frequently in males than in females. Nonetheless, the exact differences between men and women at the genetic, cellular, or functional levels of the body are largely unknown. Indeed, for some diseases, for example psychiatric disorders, differences in gender (i.e., a person's self representation as a man or woman and how that person is responded to by social institutions) might be more important than differences in sex (i.e., the classification by reproductive organs and chromosomal complement) [3]. Sex-based medicine promises to take the unique biological and physiological differences between the sexes into consideration to deliver better and more targeted health care.

Although differences in risks for and prognoses of several diseases have been well documented [4], [5], [6], [7], sex-based differences in responses to pharmaceutical treatments and accompanying risks of adverse events are less clear. Variations in absorption, distribution, metabolism, and excretion of pharmaceuticals between men and women have been investigated and demonstrated for various drugs. For example, the clearance of methylprednisolone is greater in men than in women during the late luteal cycle [8]. Similarly, isoproterenol exhibits a dose-response gradient to vasodilation in men but not in women [9]. The majority of these findings indicate differences on physiological, pharmacodynamic, or pharmacokinetic outcomes and are mostly attributed to hormonal fluctuations. Whether such findings translate into clinically relevant differences in efficacy and safety of pharmacological treatments remains undetermined.

The Drug Effectiveness Review Project (DERP) was founded in 2003 by the Center for Evidence-based Policy of the Oregon Health and Science University to provide policy-makers with the best available evidence regarding the comparative efficacy, effectiveness and safety of drugs within the same drug class [10]. Ten U.S. States and Canada currently contribute to this initiative. DERP reports are high quality systematic reviews that are standardized in methods and structure. They undergo extensive peer and public review before being finalized. To date, DERP has covered 36 commonly prescribed classes of medications. A specific feature of DERP reports is that authors are required to assess differences in the efficacy and safety of drugs in various subgroups, one of which is always sex.

The objective of our review was to determine whether clinically relevant differences in efficacy and safety exist between men and women when treated with commonly prescribed medications.

## Materials and Methods

### Drug class reviews

We retrieved the latest updates of all publicly available DERP drug class reviews up to January 2010 from the project's website [11]. We excluded one report because the population of interest was women only (hormone therapy for postmenopausal women). The drug classes covered in the 35 included reports, the year of the last update, indications of interest, and the 300 included medications are listed in table S1.

Two persons independently reviewed the subgroups chapter of each report to assess whether evidence on differential treatment effects between men and women was reported. Any relevant information with respect to sex as an effect modifier was abstracted into standardized forms and dually reviewed. Discrepancies between reviewers were resolved by consensus or by consulting a third person. If the available information in the report was incomplete or erroneous, we contacted the authors and asked for clarification.

### Individual study analysis

We retrieved and assessed full-text copies of all relevant publications identified in the subgroups chapters as providing information on the impact of sex, regardless of study design and duration. We excluded studies that were rated as having poor methodological quality by the authors of the reports as well as studies published as abstracts-only because of the limited information available on methods.

One reviewer abstracted pertinent data from each study into a standardized data abstraction form. Items of interest included: study design, number of participating men and women, characteristics of included population and indications, outcomes of interest, methods of outcomes and adverse events assessment, statistical approach regarding subgroup analyses, and results. A second reviewer cross-checked the abstracted information. Differences were resolved by consensus.

Our main outcomes of interest across all indications and drug classes were endpoints that could be viewed as health outcomes (i.e., any outcome that a patient can feel or experience). We included surrogate and intermediate outcome measures only if no relevant health outcomes were available.

### Data synthesis

We grouped the data from each drug class according to indication. If data were sufficient we calculated relative risks and 95% confidence intervals as summary statistics comparing treatment effects between men and women. If more than two studies within the same drug class examined the same outcome in a similar population, we conducted meta-analyses to achieve a pooled estimate of the effect. For each meta-analysis we ran a test of heterogeneity (I^2^ index) and applied both random and fixed effects models. We assessed publication bias by using funnel plots and Begg's adjusted correlation tests. All statistical analyses were done in StatsDirect Statistical Software program, version 2.7.7 (StatsDirect, Sale, United Kingdom).

If a particular study was included in a meta-analysis or pooled data analysis of good or fair quality, we did not incorporate this study again in the synthesis of the evidence. Where pooling was not possible we summarized the evidence qualitatively and present point estimates from the best available evidence.

### Classification of the effect of sex

We classified differences between men and women as “insignificant”, “favors men”, “favors women”, or “conflicting”. The classification “insignificant” was used for direct comparisons between men and women that did not render a statistically significant difference. If no direct comparisons were available, we assessed point estimates of stratified treatment effects. We classified differences as “insignificant” if point estimates of relative treatment effects of men and women were within a range of 25% relative risk reduction or increase. We classified the evidence as “favors men” or “favors women” when direct comparison demonstrated a statistically significant difference in efficacy or risks of adverse events or when differences in point estimates of relative treatment effects were outside a range of 25% relative risk reduction or increase. If results on different outcomes within the same study were conflicting or if studies of similar internal validity rendered contradicting findings, we classified the evidence as “conflicting”.

### Rating the quality of the evidence

Two persons rated the quality of the available evidence in a four part hierarchy (high, moderate, low, very low) based on an approach devised by the GRADE (Grading of Recommendations Assessment, Development and Evaluation) Working Group using the GRADE Profiler [12], [13]. This approach incorporates four key elements: study limitations (in design and conduct), consistency of findings of the underlying evidence, directness of its relationship to the medical practice under consideration, and precision of results. Ratings reflect the quality of the body of evidence to support or reject the notion that differences in treatment effects (beneficial or adverse) exist between men and women for a specific indication. Discrepancies in ratings were resolved by consensus.

## Results

**Figure 1:**
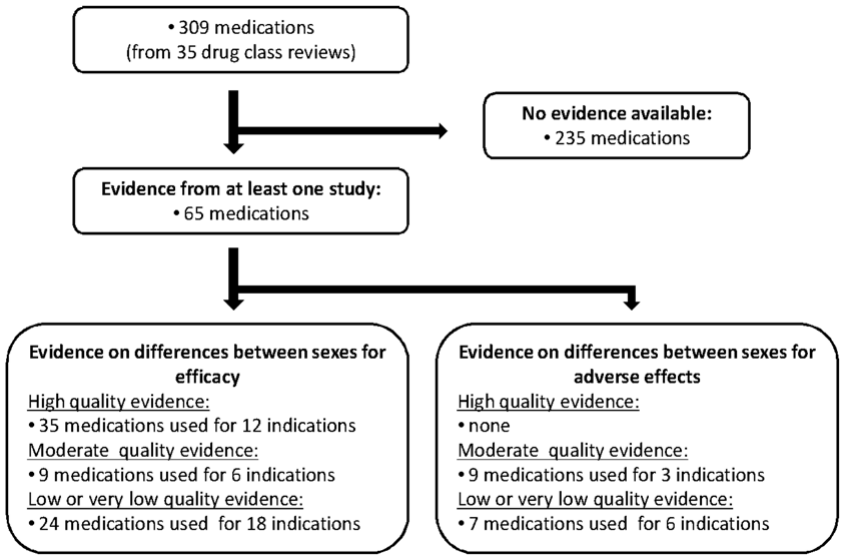
Flow of reports and publications and the number of outcomes for which high, moderate or low quality evidence was available.

Eighteen of the 35 drug class reviews identified evidence concerning the impact of sex on the efficacy and safety of drugs. Overall, we retrieved 59 studies that addressed our question of interest, including data on over 250,000 patients. These studies provide evidence on 65 individual medications (23% of all medications in the drug class reviews, see table S1). Figure 1 presents the flow of reports and publications and summarizes the number of outcomes for which high, moderate, or low/very low quality evidence was available.

For most indications the available evidence was compromised by methodological limitations. Differences in the efficacy and risks of adverse events between men and women were rarely compared directly. Most commonly, variations in treatment effects between the sexes were examined within a multitude of subgroup analyses and very few studies employed tests of interaction to determine pre-specified subgroup effects.

### The impact of sex on efficacy

In total, 53 studies assessed the impact of sex on the efficacy of medications. These studies provided information on 68 drugs used for the treatment of 36 indications. The majority of studies demonstrated similar treatment effects between men and women. With the exception of one drug class (newer antiemetic drugs), studies that reported better responses to treatment for either men or women were generally of small sample size or had methodological shortcomings.

Table S2 summarizes the available evidence, relevant outcomes, and the quality of the evidence for each drug class and relevant indications. We rated the quality of the evidence for 18 indications as low or very low which implies that results are uncertain und very likely to be changed by new studies. The evidence on drug classes for six indications was rated as moderate, suggesting that current findings are still likely to be altered by new evidence.

Seven drug classes used for the treatment of 12 indications received a rating of high-quality : (1) Angiotensin converting enzyme inhibitors (ACE-inhibitors) in patients with chronic heart failure; (2) angiotensin II receptor blockers (ARBs) in patients with chronic heart failure; (3) beta-blockers for patients with myocardial infarction and for patients with left ventricular systolic dysfunction; (4) newer antiplatelet agents for patients with coronary artery disease; (5) statins in persons with hypercholesteremia; (6) second-generation antidepressants for the treatment of major depressive disorder; and (7) newer antiemetic agents for the prevention of chemotherapy-induced nausea and emesis. A rating of high quality of evidence indicates that results are unlikely to be changed by future studies.

**Figure 2:**
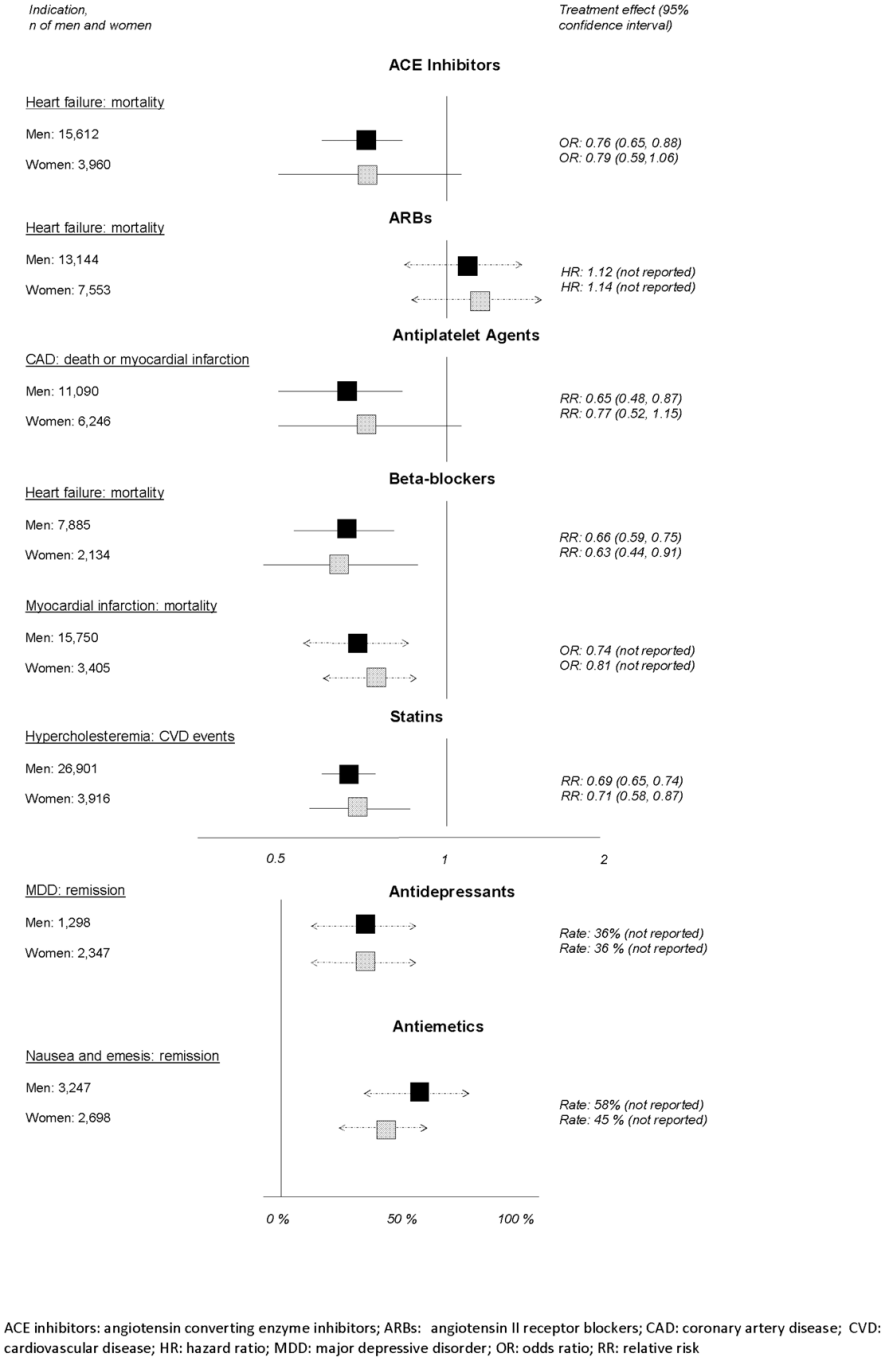
Summary of treatment effects based on high-quality evidence.

In the following paragraphs we summarize the available evidence for drugs and indications that were supported by evidence of high quality. Figure 2 depicts treatment effects for men and women of medications for which conclusions are supported by high-quality evidence.

#### Meta-analysis: Newer antiemetic agents for the prevention of chemotherapy-induced nausea and emesis

**Figure 3:**
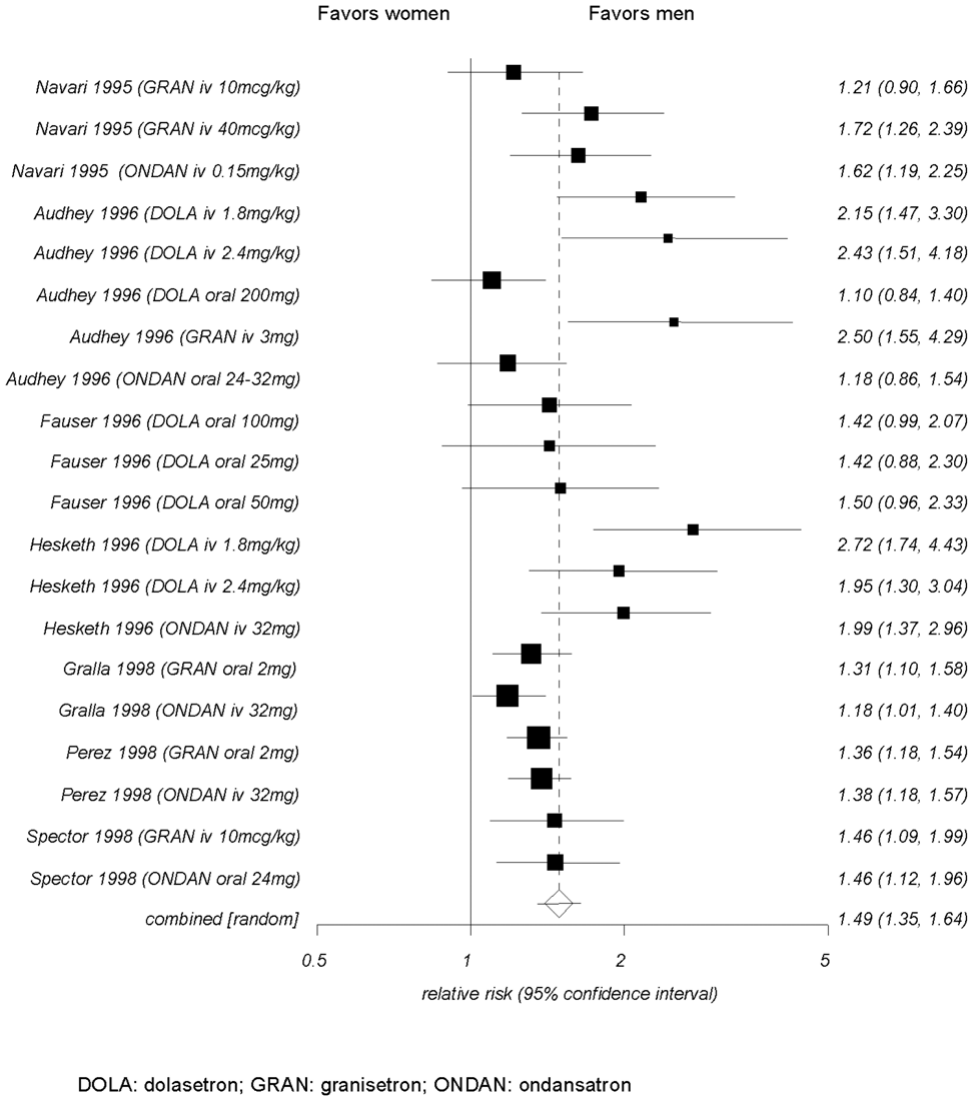
Pooled response rates of men versus women for different dosages of newer antiemetics.

Newer antiemetic agents (dolasetron, granisetron, ondansatron) is the only drug class where high-quality evidence supports differences in treatment effects between men and women. Men undergoing chemotherapy frequently responded better to prophylactic treatment with newer antiemetics than women [14], [15], [16], [17], [18], [19], [20]. We pooled response rates (prevention of nausea and vomiting) comparing men with women for different dosing regimens of dolasetron, granisetron, and ondansatron (figure 3). Results based on 20 treatment arms (seven randomized controlled trials [RCTs]) with data on more than 4900 patients indicated that, on average, 58% of men compared with 45% of women responded to treatment (relative risk 1.49, 95% CI 1.35–1.64).

#### ACE-inhibitors in patients with chronic heart failure

Two well conducted meta-analyses of ACE-inhibitors in more than 19,000 patients [21]–[22] yielded similar reductions in heart failure mortality between men (OR 0.76, 95% CI 0.65–0.88) and women (OR 0.79, 95% CI 0.59–1.06) compared with placebo [22].

#### ARBs in patients with chronic heart failure

Three subgroup analyses of male and female patients with heart failure treated with ARBs [23], [24], [25], indicated beneficial effects on mortality irrespective of sex. For example, the hazard ratio for mortality was 0.87 for men vs. 0.88 for women (test for interaction p = 0.87) [24].

#### Beta-blockers for patients with chronic heart failure or myocardial infarction

One meta-analysis of male and female patients with chronic heart failure treated with bispropolol, carvedilol, and metoprolol [26], calculated similar reductions in mortality rates for men (RR 0.66, 95% CI 0.59–0.75) and women (RR 0.63, 95% CI 0.44–0.91) compared with placebo. Likewise, beta-blockers exhibited similar benefits in male and female patients with post-myocardial infarction. Two pooled analyses of RCTs yielded similar reductions in mortality for men (OR 0.74, 95% CI not reported) and women (OR 0.81, 95% CI not reported) [27], [28].

#### Statins in persons with hypercholesteremia

Treatment effects were similar between the sexes with respect to major coronary effects and cardiovascular disease mortality in patients with hypercholesteremia treated with statins. One well-conducted meta-analysis reported a similar relative and absolute risk reduction of major coronary effects between men and women treated with statins (number needed to treat [NNT] for men 27, 95% CI 23–43; NNT for women 31, 95% CI 19–75) [29].

#### Newer antiplatelet agents for patients with coronary artery disease

Three subgroup analyses of RCTs[30], [31], [32] of patients with coronary artery disease treated with clopidogrel reported similar decreases in cardiovascular disease-mortality and myocardial infarction in men (RR 0.65, 95%CI 0.48–0.87) and women (RR 0.77, 95%CI 0.52–1.15) [32].

#### Second-generation antidepressants for major depressive disorder

A pooled analysis of eight RCTs on more than 3,500 patients with major depressive disorder detected similar remission rates for men (36%) and women (36%) treated with fluoxetine, fluvoxamine, or paroxetine [33]. Likewise, male and female patients treated with venlafaxine for major depressive disorder achieved similar remission rates (45% vs. 45%) [34].

### The impact of sex on the risk of adverse events

We included 11 studies that assessed the impact of sex on the risks of adverse events of medications. These studies provided information on 16 drugs used for the treatment of nine indications. Table S3 summarizes the quality of the available evidence for adverse events.

Methodological limitations compromised all available studies for adverse events. No high quality evidence could be identified for any of the drug classes and indications. Moderate-quality evidence indicates that men experience more sexual adverse events with paroxetine and that more women withdraw from trials due to adverse events when taking lovastatin.

Specifically, a pooled analysis of three RCTs of bupropion or paroxetine for major depressive disorder reported that men treated with paroxetine experienced higher rates of medication-related sexual dysfunction than women (change in score on the Sexual Functioning Questionnaire: men −4.14 vs. women +0.46; P  =  not reported) [35]. In comparison, a similar improvement in sexual dysfunction was reported for male and female patients treated with bupropion.

In one large trial of patients treated with lovastatin, the risk for women to discontinue treatment because of adverse events was more than twice as high as in men (OR 2.5; 95% CI 1.5–4.2) [36].

## Discussion

This umbrella review included information derived from 59 studies on more than 250,000 patients. For the majority of drugs that had available evidence, sex does not appear to be a factor that has to be taken into consideration when choosing a drug treatment. Three clinically relevant exceptions, however, are evident. Men achieved substantially better response rates than women when treated with newer antiemetic drugs. This information might be valuable to clinicians in anticipating the need to move to second-line therapy in women who do not respond to recommended prophylaxis for nausea and vomiting while undergoing chemotherapy. Likewise, treatment with paroxetine led to substantially higher rates of sexual dysfunction in men than in women, while no differences could be observed between male and female patients treated with bupropion. Given similar response and remission rates to second-generation antidepressants [37], [38], the higher risk of sexual dysfunction for men treated with paroxetine should be taken into consideration when choosing an antidepressant. Clinicians should also be aware that women are more likely than men to discontinue statins due to adverse events.

Our study has some limitations. The available body of evidence for most drug classes was limited in quality and quantity, confining the range and the certainty of our conclusions. Only few results are supported by high-quality evidence that is reliable enough to assume that future studies will not change the estimates of the effects. In particular, the methods of subgroup analyses were often inadequate. They were typically conducted post hoc, sometimes without correcting for multiple testing, increasing the risk of false findings and incorrect conclusions [39]. Similarly, most studies were not large enough to provide the statistical power to detect differences between men and women.

In addition, in many trials the focus is on statistically significant findings. Authors of studies may have chosen not to report subgroup analyses by sex that did not yield statistically significant differences (outcome reporting bias). In this case, our findings would be distorted towards the existence of differences between men and women, which was not the case.

We relied on completed drug class reviews and did not reproduce the literature searches for each report. Authors of these reviews may have incompletely summarized the evidence on sex. We believe that it is unlikely that any missed studies would have changed our conclusions because the methods of DERP include an extensive peer and public review process. Nonetheless, it is possible that new studies with relevant information have been more recently published than the drug class reviews. For example, a recent trial of statin therapy for primary prevention of cardiovascular disease with more than17,000 participants did not demonstrate a difference in the rate of adverse events between men and women, which contrasts with our findings [40].

The scope of our review is limited to commonly prescribed drugs that are of economic importance to participating organizations of the DERP project. Although a large variety of drugs have been covered, some commonly used medications have not been included. For some of these drugs, such as aspirin, evidence suggests no clinically important differences in treatment effects [41].

Although most of the results derived from high-quality evidence indicate that differences in efficacy and risks of adverse events are negligible, findings are not generalizable to other drug classes or different indications.

Categorizing patients as “men” and “women“ is based on differences in societal roles, behavioral responses, risks for certain diseases, and life expectancies. Nevertheless, neither men nor women are homogenous groups and genetic variations between and within the sexes may have a more important impact on response to drug therapy than sex alone [42]. With the advance of pharmacogenetics, future studies need to take relevant genetic variations as potential confounders into consideration when determining differences in efficacy and risks for adverse events between men and women.

## Acknowledgments

We would like to thankfully acknowledge Maria Wieser and Stephanie Gattinger for help with data abstraction and Irene Wild for support with formatting the paper.

## Supporting Information

Table S1 Overview of indications and medications of included drug class reviews.
(0.10 MB DOC)

Table S2 Evidence profile of the impact of sex on the efficacy of drugs.
(0.28 MB DOC)

Table S3 Evidence profile of the impact of sex on the risk of adverse events of drugs.
(0.08 MB DOC)
