# Supplementary material for: Differences in Efficacy and Safety of Pharmaceutical Treatments between Men and Women: An Umbrella Review
Source: PLoS One. 2010 Jul 30;5(7):e11895. doi: 10.1371/journal.pone.0011895 (PMC2912767; doi:10.1371/journal.pone.0011895)
Supplement: Table S1 — Overview of indications and medications of included drug class reviews. (0.10 MB DOC) [file pone.0011895.s001.doc]

Table S1: Overview of Indications and Medications of Included Drug Class Reviews

| **Drug class,**  **last update** | **Indications of interest** | **Medications included in drug class review** | **Medications with evidence on the effect of sex on efficacy and risks of adverse events** |
| --- | --- | --- | --- |
| Alzheimer's Drugs,[43] June 2006 | Alzheimer's Disease | donepezil, galantamine, memantine, rivastigmine, tacrine | rivastigmine |
| Angiotensin Converting Enzyme Inhibitors,[44] June 2006 | Essential Hypertension, Heart Failure, High Cardiovascular Risk Factors, Diabetic Nephropathy, Nondiabetic Nephropathy, Recent Myocardial Infarction | benazepril, captopril, cilazapril, enalapril, fosinopril, lisinopril, moexipril, perindopril, quinapril, ramipril, trandolapril | benazepril, captopril, cilazepril, enalapril, lisinopril, perindopril, qinapril, ramipril, trandolopril, zofenopril, |
| Angiotensin II Receptor Antagonists,[45] February 2006 | Essential Hypertension, Heart Failure, High Cardiovascular Risk Factors, Diabetic Nephropathy, Nondiabetic Nephropathy | candesartan, eprosartan, irbesartan, losartan, olmesartan, telmisartan, valsartan | candesartan, losartan, valsartan, |
| Second Generation Antidepressants,[46] October 2008 | General Anxiety Disorder, Depressive Disorder, Adjustment Disorder, Dysthymic Disorder, Subsyndromal Disorder, Season Affective Disorder, Obsessive Compulsive Disorder (OCD), Panic Disorder, Post-Traumatic Stress Disorder (PTSD), Social Anxiety Disorder, Premenstrual Dysphoric Disorder (PMDD) | bupropion, citalopram, duloxetine, escitalopram, fluoxetine, fluvoxamine, mirtazapine, nefazodone, paroxetine, sertraline, venlafaxine | bupropion, duloxetine, fluoxetine, fluvoxamine, paroxetine, sertraline venlafaxine |
| Antiepileptic Drugs for Indication other than Epilepsy,[47] October 2008 | Bipolar Disorders, Chronic Pain, Fibromyalgia, Migraine | carbamazepine, divalproex sodium, ethotoin, gabapentin, lamotrigine, levetiracetam, oxcarbazepine, phenytoin, pregabalin, tiagabine, topiramate, valproic acid, zonisamide | divalproex, gabapentin, lamotrigine, lithium |
| Newer Antiemetics,[48] January 2009 | Nausea, Vomiting | aprepitant/fosaprepitant, dolasetron, granisetron, ondansetron, palonosetron | dolasetron, granisetron, ondansetron |
| Newer Antihistamines,[43] April 2006 | Seasonal Rhinitis, Perennial Rhinitis, Urticaria | cetirizine hydrochloride, desloratadine, fexofenadine hydrochloride, loratadine | none |
| Newer Antiplatelets Agents,[49] April 2007 | Coronary Artery Disease, Coronary Revascularization, Myocardial Infarction, Acute Coronary Syndrome | aspirin 25 mg/ dipyridamole er 200 mg, clopidogrel, dipyridamole, ticlopidine | clopidegrel, dipyridamole, ticlopedone |
| Atypical Antipsychotics,[50] June 2008 | Bipolar Disorder, Dementia, Alzheimer’s, Schizophrenia, Schizophreniform Disorder, Delusional Disorder, Autistic Disorder, Rett Disorder, Childhood Disintegrative Disorder, Asperger Disorder, Pervasive Developmental Disorder, Atypical Autism, Oppositional Defiant Disorder, Conduct Disorder, Disruptive behavior Disorder | aripiprazole, clozapine, olanzapine, quetiapine, risperidone, ziprasidone | olenzapine plus lithium or valproate, lithium, risperidone, valproate, |
| Controller Medications for Asthma,[51] November 2008 | Persistent Asthma | arformoterol, beclomethasone dipropionate, budesonide, budesonide/formoterol, flunisolide, fluticasone propionate, fluticasone propionate/salmeterol xinafoate, formoterol fumarate/eformoterol, mometasone furoate, montelukast, omalizumab, salmeterol xinafoate, triamcinolone acetonide, zafirlukast, zileuton | none |
| Quick Relief Medications for Asthma,[52] October 2008 | Bronchospasm due to Asthma, Exercise-Induced Bronchospasm | albuterol, fenoterol, ipratropium bromide, ipratropium bromide and albuterol sulfate, levalbuterol, pirbuterol, terbutaline | none |
| Pharmacologic Treatments for Attention Deficit Hyperactivity Disorder,[53] October 2009 | Attention Deficit Hyperactivity Disorder | amphetamine mixture (amphetamine aspartate; amphetamine sulfate; dextroamphetamine saccharate; dextroamphetamine sulfate), atomoxetine hcl, dexmethylphenidate hydrochloride, dextroamphetamine sulfate, lisdexamfetamine dimesylate, methamphetamine hydrochloride, methylphenidate hydrochloride, modafinil | atomexetine, dextroamphetamine, metadate cd/equasym xl, methylphenidate |
| Beta Adrenergic Blockers,[54] July 2009 | Angina, Atrial Arrhythmia, Coronary Revascularization, Esophageal Varices, Heart Failure, Hypertension, Migraine, Myocardial Infarction | acebutolol, atenolol, betaxolol, bisoprolol, carteolol, carvedilol, carvedilol phosphate, labetalol, metoprolol tartrate, metoprolol succinate, nadolol, nebivolol, penbutolol, pindolol, propranolol, propranolol long-acting, timolol | alprenolol, bisoprolol, carvedilol, metoprolol, nebivolol, oxpranolol, pindolol, practolol, propranolol, sotalol, timolol |
| Beta2 Agonists,[55] November 2006 | Asthma, Chronic Obstructive Pulmonary Disease, Exercise Induced Bronchospasm | albuterol, fenoterol, formoterol, levalbuterol, metaproterenol, pirbuterol, salmeterol, terbutaline | none |
| Calcium Channel Blockers,[56] March 2005 | Angina, Essential Hypertension, Supraventricular Arrhythmias | amlodipine, diltiazem, isradipine, nicardipine, nifedipine gits, nifedipine retard, nisoldipine, coerverapamil, verapamil sr | none |
| Constipation Drugs,[57] September 2007 | Constipation, Chronic Constipation Associated with Irritable Bowel Syndrome | docusate calcium, docusate sodium, lactulose, lubiprostone, polyethylene glycol 3350, psyllium (ispaghula), tegaserod maleate | none |
| Cyclo-oxygenase (COX)-2 Inhibitors and Non-steroidal Anti-inflammatory Drugs (NSAIDs),[58] November 2006 | Ankylosing Spondylitis, Back Pain, Osteoarthritis  Rheumatoid Arthritis, Soft-tissue pain, Dysmenorrhea, Acute Pain (Dental or Surgical Pain) | celecoxib, diclofenac sodium, diclofenac potassium, diflunsial, etodolac, fenoprofen, flurbiprofen, ibuprofen, indomethacin, ketoprofen, ketoprofen xr, ketorolac, meclofenamate, mefenamic acid, meloxicam, nabumetone, naproxen, naproxen delayed release, naproxen sodium, oxaprozin, piroxicam, salsalate, sulindac, tiaprofenic acid, tenoxicam, tolmetin | none |
| Fixed Dose Combination Products for Diabetes Mellitus and Hyperlipidemia,[59] October 2007 | Type 2 Diabetes, Hyperlipidemia | ezetimibe/simvastatin, glipizide/ metformin , glyburide/ metformin, niacin/lovastatin, pioglitazone/glimepiride, pioglitazone/metformin, rosiglitazone/ glimepiride, rosiglitazone/metformin, sitagliptin/metformin | lovastatin, niacin extended release |
| Newer Drugs for the Treatment of Diabetes Mellitus,[60] August 2008 | Type 1 Diabetes, Type 2 Diabetes | exenatide, pramlintide, sitagliptin | sitagliptin |
| Inhaled Corticosteroids,[61] January 2006 | Asthma, Chronic Obstructive Pulmonary Disease | beclomethasone dipropionate, budesonide, flunisolide, fluticasone propionate, mometasone furoate, triamcinolone acetonide | none |
| Newer Drugs for Insomnia,[62] October 2008 | Insomnia, Sleep Disorder | eszopiclone, ramelteon, zaleplon, zopiclone, zolpidem, zolpidem extended-release | none |
| Pegylated Interferons for Chronic Hepatitis C Infection,[63] May 2007 | Chronic Hepatitis C Virus Infection | interferon monotherapy, interferon plus ribavirin, pegylated interferon monotherapy, pegylated interferon plus ribavirin | none |
| Disease-modifying drugs for Multiple Sclerosis,[64] July 2007 | Multiple Sclerosis | glatiramer acetate, interferon β 1a (avonex®), interferon β 1a (rebif®), interferon β 1b, mitoxantrone, natalizumab | none |
| Nasal Corticosteroids,[65] June 2008 | Seasonal Rhinitis, Perennial Rhinitis | beclomethasone, budesonide, ciclesonide, flunisolide, fluticasone furoate, fluticasone propionate, mometasone, triamcinolone | none |
| Drugs for Neuropathic Pain,[66] October 2007 | Neuropathic Pain | amitriptyline, carbamazepine, citalopram, desipramine, dextromethorphan, doxepin, duloxetine, escitalopram, fluoxetine, gabapentin, imipramine, lamotrigine, lidocaine patch 5%, lidocaine topical gel 5%, nortriptyline, oxcarbazepine, paroxetine, pregabalin, sertraline, topiramate, valproic acid/divalproex, venlafaxine | none |
| Long-Acting Opioid Analgesics,[67] April 2008 | Chronic Non-Cancer Pain | oxycodone, morphine, methadone, fentanyl, levorphanol, codeine, dihydrocodeine, oxymorphone | none |
| Oral Hypoglycemics[68] May 2005 | Type 2 Diabetes | chlorpropamide, glimepiride, glipizide, glyburide, glyburide micronized, nateglinide, repaglinide, tolazamide, tolbutamide | none |
| Agents for Overactive Bladder,[69] March 2009 | Overactive Bladder, Urinary Incontinence | butylbromide, darifenacin, flavoxate hydrochloride, hyoscyamine sulfate, oxybutynin chloride, oxybutynin, scopolamine (hyoscine), solifenacin succinate, tolterodine tartrate, trospium chloride | tolterodine |
| Proton Pump Inhibitors,[70] May 2009 | Gastroesophageal Reflux Disease, Peptic Ulcer and Nonsteroidal Anti-inflammatory, Drug-induced ulcer, Helicobacter pylori infection | esomeprazole, lansoprazole, omeprazole, omeprazole/sodium bicarbonate, pantoprazole, rabeprazole, | esomeprazole, omeprazole, rabeprazole, |
| Skeletal Muscle Relaxants,[71] May 2005 | Traumatic Brain Injury, Cerebral Palsy, Fibromyalgia, Tension Headaches, Multiple Sclerosis, Myofascial Pain Syndromes, Nocturnal Leg Cramps, Mechanical Low Back or Neck Pain, Spinal Cord Injury, Stroke Post-stroke Syndrome | baclofen, carisoprodol, chlorzoxazone, clonazepam, clonidine, clorazepate, cyclobenzaprine, dantrolene, diazepam, gabapentin, metaxalone, methocarbamol, orphenadrine, quinine, tizanidine | none |
| Statins-HMG-CoA Reductase Inhibitors,[72] November 2009 | Coronary Artery Disease, Myocardial Infarction, Revascularization, Stroke | atorvastatin, fluvastatin, lovastatina, pravastatina, rosuvastatin, simvastatina, lovastatin/niacin-er, simvastatin/niacin-er, simvastatin/ezetimibe | atorvastatin, lovastatin, pravastatin, simvastatin, |
| Targeted Immune Modulators,[73] November 2009 | Ankylosing Spondylitis, Crohn's Disease, Plaque Psoriasis, Psoriatic Arthritis, Rheumatoid Arthritis, Ulcerative Colitis, Juvenile Idiopathic Arthritis | abatacept, adalimumab, alefacept, anakinra, certolizumab pegol, efalizumab, etanercept, infliximab, natalizumab, rituximab | adalimumab, etanercept, infliximab |
| Thiazolidinediones,[74] August 2008 | Diabetes, Metabolic Syndrome, Prediabetes | pioglitazone, rosiglitazone, | none |
| Topical Calcineurin Inhibitors,[75] October 2008 | Atopic Dermatitis, Eczema | tacrolimus, pimecrolimus | none |
| Triptans,[76] June 2009 | Migraine |  | none |
