# Supplementary material for: Differences in Efficacy and Safety of Pharmaceutical Treatments between Men and Women: An Umbrella Review
Source: PLoS One. 2010 Jul 30;5(7):e11895. doi: 10.1371/journal.pone.0011895 (PMC2912767; doi:10.1371/journal.pone.0011895)
Supplement: Table S2 — Evidence profile of the impact of sex on the efficacy of drugs. (0.28 MB DOC) [file pone.0011895.s002.doc]

Table S2: Evidence Profile of the Impact of Sex on the Efficacy of Drugs

| **Number of Studies, Study Design** | **No of Patients** | | **Population & Indication** | **Limitations** | **Outcomes assessed** | **Consistency of Results** | **Impact of Sex on Treatment Effect** | **Quality of the Evidence*** |
| --- | --- | --- | --- | --- | --- | --- | --- | --- |
| **Alzheimer’s Drugs** | | | | | | | | |
| 1 pooled analysis of 3 RCTs[77] | 878 | 1,248 | Patients with Alzheimer’s type dementia and a MMSE between 10 and 26 treated with rivastigmine | Some^1,2^ | Cognition, daily function, memory and behavior | N/A | **Insignificant**  Similar treatment effects between men and women (data NR) | Very low |
| **Angiotensin Converting Enzyme Inhibitors** | | | | | | | | |
| 2 meta-analyses of RCTs[21,22] | >10,000 | >2,300 | Patients with heart failure (NYHA class II-III) or reduced LVEF treated with enalapril, zofenopril, ramipril, trandolopril, captopril, benazepril, cilazepril, lisinopril, perindopril, quinapril. | None | Total mortality and mortality from heart failure | Yes | **Insignificant**  Similar reduction in heart failure mortality rates for men (OR 0.76, 95% CI 0.65-0.88) and women (OR 0.79, 95% CI 0.59-1.06) compared with controls | High |
| **Angiotensin II Receptor Antagonists** | | | | | | | | |
| 3 subgroup analyses of RCTs[23,24,25] | 13,144 | 7,553 | Patients with heart failure (NYHA class II-IV) with/without reduced LVEF treated with candesartan, valsartan, losartan | Some^2^ | Mortality  & morbidity (cardiac arrest, worsening heart failure, stroke) | Yes | **Insignificant**  Similar reduction in mortality across subgroup sex, men HR 0.87, women 0.88 (test for interaction p=0.87) | High |
| 1 subgroup analyses of an RCT[78] | 1,753 | 3,184 | Patients with hypertension treated with candesartan | Some^2^ | Major cardiovascular event (CVD-death, MI, or stroke) | N/A | **Insignificant**  Similar reduction in CV events between men (RR 0.86, 95%CI 0.67-1.12) and women (RR 0.92 95%cI 0.72-1.17) compared with placebo | Very low |
| **Second Generation Antidepressants** | | | | | | | | |
| 1 pooled analysis of RCTs[33,34] | 1,298 | 2,347 | Patients with major depressive disorder treated with fluoxetine, fluvoxamine, paroxetine, venlafaxine | Some^1^ | Response, remission | N/A | **Insignificant**  Similar remission rates for SSRIs (36% vs. 36%; P >0.05) and venlafaxine (45% vs. 45%, P >0.05) | High |
| 1 pooled analysis of RCTs[79] | 335 | 338 | Patients with panic disorder treated with sertraline | Serious^1,2^ | Response, frequency of panic attacks, quality of life | N/A | **Insignificant**  Similar response to treatment (CGI-I 2.32 vs. 2.19; p =0.32) and panic disorder severity (PDSS change -5.79 vs. -6.99; p = 0.12) | Low |
| **Antiepileptic Drugs for Indications Other Than Epilepsy** | | | | | | | | |
| 1 subgroup analysis of RCT[80] | 18 | 27 | Patients with treatment refractory bipolar or unipolar affective disorder treated with lamitrogine and gabapentin | Some^2^ | Overall improvement on the CGI-BP (response) | N/A | **Favors men**  Significant relationship between the degree of lamotrigine response and the male gender (data NR) | Very Low |
| 1 subgroup analysis of RCT[81] | NR | NR | Patients with bipolar disorder type I treated with divalprolex | Some^2^ | Depressive relapse | N/A | **Favors men**  Men were less likely than women to experience depressive relapse (OR= 0.51 95%CI 0.31-0.82) | Very Low |
| **Newer Antiemetics** | | | | | | | | |
| 8 subgroup analyses of RCTs[14,15,16,17,18,19,20,82] | 3,247 | 2,698 | Adult patients scheduled to receive cisplatin or other emetogenic chemotherapy (with or without corticosteroids) treated with ondansetron, granisetron, dolasetron | Some^1,2^ | Control of nausea and emesis, no use of rescue medication | Yes | **Favors men**  Higher rates of complete control of nausea and emesis in men than in women (58% vs. 45%; relative risk 1.48, 95% CI 1.35-1.62). | High |
| **Newer Antiplatelet Agents** | | | | | | | | |
| 3 subgroup analyses of RCTs[30,31,32] | 11,090 | 6,246 | Patients with CAD, Coronary ischemia or ACS without ST-segment elevation treated with clopidegrel | Some^2^ | CVD-mortality, MI & stroke | Yes | **Insignificant**  Similar reduction in CVD-death or MI in men and women, men RR 0.65 (95%CI 0.48 to 0.87) and women RR 0.77 (95%CI 0.52 - 1.15) | High |
| 2 subgroups analyses of RCTs[83,84] | >1,900 | >1,000 | Patients with a history of stroke or TIA treated with ticlopedone or dipyridamole | Some^2^ | Mortality and stroke, or TIA | Yes | **Insignificant**  Similar reduction of the risk of stroke or death between men and women: 3-year risk reduction for men 10% (95%CI -8 to 27) and women 17% (95%CI -7 to 41) | Moderate |
| **Pharmacological Treatments for ADHD** | | | | | | | | |
| 2 RCTs,[85,86] 2 subgroup analyses of RCTs,[87,88] & 1 observational study[89] | >220 | >120 | Children with ADHD, learning disorder, or conduct disorder treated with methylphenidate, dextroamphetamine, Metadate CD/Equasym XL | Some^2^ | Behavior, conduct and mother-child interaction, interaction and attention (SKAMP scale) | No | **Insignificant**  Similar response for boys and girls; however the improvement in boys lasted longer than in girls | Low |
| 1 subgroup analysis of 2 pooled RCTs[90] | 348 | 188 | Adults with ADHD treated with atomexetine | Some^2^ | ADHD-specific adult scores (CAARS-INV & WRAADDS), Sheehan Disability Scale, CGI-S, & HAM-D/HAM-A | N/A | **Insignificant**  No significant treatment-by-gender interaction for WRAADDS (F=1.22, df=1,446; p=0.269) CAARS-INV (F=0.617, df=1,511; p=0.433) or for Sheehan Disability Scale, CGI-S, HAM-D or HAM-A | Low |
| **Atypical Antipsychotic Drugs** | | | | | | | | |
| 1 subgroup analysis of RCT[91] | 147 | 115 | Patients with bipolar I disorder, current episode mania treated with risperidone monotherapy | Serious^2^ | Response to treatment (Young Mania Rating Scale) | N/A | **Insignificant**  Similar response to treatment for men and women (data NR) | Low |
| 1 subgroup analysis of RCT[92] | 48 | 51 | Patients with bipolar I disorder and current syndromic remission treated with olenzapine plus lithium or valproate, or lithium or valproate as monotherapies | Some^1^ | Relapse prevention (DSM-IV, and Hamilton Depression Rating Scale) | N/A | **Favors men**  Time to relapse was similar in men treated with mono- or combination therapy (67 vs. 84 days); women had a statistically significantly shorter time to relapse on mono- than on combination therapy (27.5 vs. 177 days; P = 0.001) | Low |
| **Beta Adrenergic Blockers** | | | | | | | | |
| 1 meta-analysis of RCTs[26] | 7,885 | 2,134 | Patients with heart failure and left ventricular systolic dysfunction treated with bisoprolol, carvedilol, and metoprolol | None | Mortality | N/A | **Insignificant**  Similar reduction in mortality rates for men (RR 0.66, 95% CI 0.59-0.75) and women (RR 0.63, 95% CI 0.44-0.91) compared with placebo | High |
| 1 subgroup analysis of RCT[93] | 1,343 | 785 | Elderly patients with heart failure and left ventricular systolic dysfunction treated with nebivolol | Some^2^ | Mortality or cardiovascular hospital admission | N/A | **Insignificant**  Similar reduction in mortality rates for men (HR 0.93, 95% CI 0.78-1.11) and women (HR 0.72, 95% CI 0.55-0.93) compared with placebo (test for interaction: P =0.11) | Moderate |
| 2 pooled analysis of RCTs[27,28] | 15,750 | 3,405 | Patients with myocardial infarction treated with alprenolol, metoprolol, oxpranolol, pindolol, practolol, propranolol, sotalol, and timolol, | Some^1^ | Mortality | Yes | **Insignificant**  Similar reduction in mortality rates for men (OR 0.74, 95% CI NR) and women (OR 0.81, 95% CI NR) compared with placebo | High |
| **Fixed Dose Combination Products for Diabetes Mellitus and Hyperlipidemia** | | | | | | | | |
| 1 subgroup analysis of an RCT[94] | 130 | 106 | Patients with type II A or type II B hyperlipidemia treated with niacin extended release and/or lovastatin | Some^2^ | Changes in lipoprotein levels | N/A | **Favors women**  Changes in lipid parameters with niacin-containing regimens were lower in men than women (data NR) | Low |
| 2 subgroup analysis of RCTs[95,96] | 754 | 753 | Patients with type 2 diabetes who have failed to achieve glycaemic control despite diet, exercise , and sulphonylurea therapy treated with glyburide, metformin or  glipizide/metformin | Some^1,2^ | Changes in HbA_1c_ | Yes | **Insignificant**  Similar changes in HbA1c between men and women (data NR) | Moderate |
| **Newer Drugs for the Treatment of Diabetes Mellitus** | | | | | | | | |
| 4 subgroup analyses of RCTs[97,98,99,100] | 627 | 509 | Patients with type 2 diabetes with or without metformin therapy treated with sitagliptin | Some^2^ | Changes in HbA_1c_ | Yes | **Insignificant**  Similar reduction of HbA_1c_ between men and women (change from baseline in HbA1c% for men sitagliptin -0.9 ±0.2 vs. placebo 0.0 ±0.2 and women sitagliptin -1.1 ±0.2 vs. placebo 0.1 ±0.2) | Moderate |
| **Agents for Overactive Bladder** | | | | | | | | |
| 1 post hoc data-analysis of an uncontrolled before-after study[101] | 511 | 1,711 | Patients with overactive bladder treated with tolterodine | Serious^2^ | Global efficacy, incontinence, urgency, frequency | N/A | **Conflicting**  Lower global efficacy (OR 0.67; 95% CI 0.53-0.82) and successful treatment of urgency (OR 0.76; 95% CI 0.58-1.00) and frequency (OR 0.75; 95% CI 0.55-1.00) for men vs. women; however greater improvement of incontinence for men vs. women (OR 1.45, 95% CI 1.06-1.99) | Very low |
| **Proton Pump Inhibitors** | | | | | | | | |
| 1 subgroup analysis of RCT[102] | 1,174 | 786 | Patients with reflux oesophagitis treated with esomeprazole or omeprazole | Some^2^ | Healing rates (endoscopically confirmed), heartburn resolution (4-point scale) | N/A | **Insignificant**  Similar healing rates with respect to sex (data NR) | Moderate |
| **Statins - HMG-CoA Reductase Inhibitors** | | | | | | | | |
| 1 meta-analysis,[29] 1 subgroup analysis of an RCT[103,104] | 42,322 | 9,031 | Patients with hypercholesteremia, treated with simvastatin, pravastatin, and lovastatin | None | Major coronary events, cardiovascular and non-cardiovascular death, and all-cause mortality | Yes | **Insignificant**  Similar reductions of major coronary events between men and women. NNT for men 27 (23-34), women 31 (19-75) | High |
| 1 subgroup analysis of an RCT[105] | 619 | 272 | Patients between 65 and 85 years with CAD, hypercholesteremia, and myocardial ischemia treated with atorvastatin or pravastatin | Some^2^ | Change in duration of myocardial ischemia & major acute cardiovascular events | N/A | **Insignificant**  Men experienced a smaller decrease in myocardial ischemia compared with women; however this difference did not reach statistical significance | Very Low |
| 1 pooled analysis of 4 RCTs,[106] 1 subgroup analysis of an RCT (women only)[36] | 5,949 | 4,212 | Patients with hypercholesteremia treated with simvastatin or lovastatin | Some^1,2^ | Improvement in lipids (LDL, TC, HDL, LDL/HDL ratio, LDL/TC ratio, TGs) | Yes | **Insignificant**  Lipid-lowering efficacy was similar in men and women (data NR) | Low |
| **Targeted Immune Modulators** | | | | | | | | |
| 1 Prospective cohort study[107] | 353 | 1,212 | Patients with active rheumatoid arthritis initiating the first course of treatment with adalimumab, etanercept, or infliximab | Some^1^ | Response to treatment (ACR 20 – 70%, EULAR, 28-joint Disease Activity Score) | N/A | **Insignificant**  Similar response and remission rates between men and women (data NR) | Low |
| **Newer Antihistamines, Quick-relief Medications for Asthma, Beta2-Agonists, Calcium Channel Blockers, Constipation Drugs, Controller Medications for Asthma, Cyclo-oxygenase (COX)-2 Inhibitors and Non-steroidal Anti-inflammatory Drugs (NSAIDs), Inhaled Corticosteroids, Newer Drugs for Insomnia, Pegylated Interferons for Chronic Hepatitis C Infection, Disease-modifying drugs for Multiple Sclerosis, Nasal Corticosteroids, Drugs for Neuropathic Pain, Long-Acting Opioid Analgesics, Oral Hypoglycemics, Skeletal Muscle Relaxants, Thiazolidinediones, Topical Cacineurin Inhibitors, Triptans** | | | | | | | | |
| No evidence | | | | | | | | |

^1^ Publication bias is likely

^2^ Statistical limitations that could cause inferential error (e.g.no correction for multiple testing, no test of interaction, low power for subgroup analyses, not adjusted for confounders, no control group, post hoc data-analysis, no intention-to-treat population)

^3^ Limited to patient reported adverse effects

*High: Further research is very unlikely to change our confidence in the estimate of the effect; Moderate: Further research is likely to have an important impact on our confidence in the estimate of the effect and may change the estimate; Low: Further research is very likely to have an important impact on our confidence in the estimate of the effect and is likely to change the estimate; Very low: Any estimate of effect is very uncertain

ACR: American College of Rheumatology; ACS: acute coronary syndrome; ADHD: Attention Deficit Hyperactivity Disorder; AE: Adverse Effects; CAARS-INV: Connors’ Adult ADHD Rating Scale; CAD: Coronary artery disease; CGI-BP: Clinical Global Impression Scale modified for Bipolar Illness; CGI-I: Clinical Global Impression-Improvement; CGI-S: Clinical Global Impression Scale; CI: confidence interval; CV: cardiovascular; CVD: cardiovascular disease; EULAR: European League against Rheumatism criteria; HAM-A: Hamilton Rating Scale for Anxiety; HAM-D: Hamilton Rating Scale for Depression; HDL: high-density lipoprotein; HR: hazard ratio; LDL: low-density lipoprotein; LVEF: Left Ventricular Ejection Fraction; MI: myocardial infarction; MMSE: Mini-Mental State Examination; NNT: Number Needed to Treat; NR: Not Reported; NYHA: New York Heart Association; OR: Odds Ratio; PDSS: Panic Disorder Severity Scale; RR: Relative Risk; SKAMP: Swanson, Kotkin, Agler, M-Flynn, and Pelham teacher rating scale; TC: total cholesterol; TG: Triglycerides; TIA: transient ischemic attack; WRAADDS: Wender-Reimherr Adult Attention Deficit Disorder Scale.
