# Supplementary material for: Differences in Efficacy and Safety of Pharmaceutical Treatments between Men and Women: An Umbrella Review
Source: PLoS One. 2010 Jul 30;5(7):e11895. doi: 10.1371/journal.pone.0011895 (PMC2912767; doi:10.1371/journal.pone.0011895)
Supplement: Table S3 — Evidence profile of the impact of sex on the risk of adverse events of drugs. (0.08 MB DOC) [file pone.0011895.s003.doc]

Table S3: Evidence Profile of the Impact of Sex on the Risk of Adverse Events of Drugs

| **Number of Studies, Study Design** | **No of Patients** | | **Population** | **Limitations** | **Outcomes assessed** | **Consistency of Results** | **Impact of Sex on the Risk of Adverse Events** | **Quality of the Evidence*** |
| --- | --- | --- | --- | --- | --- | --- | --- | --- |
| **Second Generation Antidepressants** | | | | | | | | |
| 1 pooled analysis of RCTs[34] | 734 | 1311 | Patients with major depressive disorder treated with fluoxetine, fluvoxamine, paroxetine, venlafaxine | Some^1^ | Frequency of adverse effects | N/A | **Conflicting**  Similar frequencies of adverse effects between men and women for: headache (men 15-26% vs. women 21-23%); insomnia (men 13-16% vs. women 13-14%); and dizziness (men 7-13% vs. women 9-13%). Men experienced less nausea than women (men 16-20% vs. women 21-28%)(p=NR). | Moderate |
| 2 pooled analyses of RCTs[79,108] | 653 | 916 | Patients with major depressive disorder treated with duloxetine and patients with panic disorder treated with sertraline | Some^1^ | Frequency of adverse effects and discontinuation rates due to adverse effects | Yes | **Insignificant**  Similar discontinuation rates because of adverse effects (18.6% vs. 13.5%; P = NR). Overall, similar frequencies of adverse effects between men and women | Moderate |
| 1 pooled analysis of RCTs[35] | 73 | 68 | Patients with major depressive disorder treated with bupropion or paroxetine | Some^1^ | Sexual dysfunction | N/A | **Conflicting**  Higher rates of medication-related sexual dysfunction for men treated with paroxetine (Sex FX change men -4.16 vs. women +2.32) not seen for bupropion (men +1.79 vs. women +0.46) | Moderate |
| **Fixed Dose Combination Products for Diabetes Mellitus and Hyperlipidemia** | | | | | | | | |
| 1 subgroup analysis of an RCT[94] | 130 | 106 | Patients with type II A or type II B hyperlipidemia treated with niacin extended release and/or lovastatin | Serious^2,3^ | Incidence of adverse effects and changes in lab values | N/A | **Favors men**  Main adverse effects (rash, flushing, headache, pruritus) were reported less commonly by men than women (data NR) | Low |
| **Agents for Overactive Bladder** | | | | | | | | |
| 2 post hoc data-analysis of an RCT[109] and an uncontrolled before-after study[101] | 12,339 | 26,876 | Patients with overactive bladder treated with tolterodine | Serious^1, 2, 3^ | Global tolerability, discontinuation due to adverse effects | Yes | **Insignificant**  Similar global tolerability (OR 0.99, 95% CI 0.79-1.25) and discontinuation rates due to adverse effects (3% vs. 3%) | Low |
| 1 uncontrolled cohort study based on prescription event monitoring[110] | NR | NR | Patients with overactive bladder prescribed tolterodine | Serious^2, 3^ | Any new event, deterioration, or reason for discontinuation, adverse reactions | N/A | **Favors men**  Statistically significant association of sex with hallucinations: men 1/11,083 vs. women 8/24,212 (P=0.013).  **Favors women**  Statistically significant association of sex with cardiac events: men 16/11,083 vs. women 20/24,212 (P<0.001).  Absolute event rates were low. | Very low |
| **Proton Pump Inhibitors** | | | | | | | | |
| 1 subgroup analysis of RCT[102] | 1,174 | 786 | Patients with reflux oesophagitis treated with esomeprazole or omeprazole | Serious^2,3^ | Incidence of adverse effects | N/A | **Insignificant**  Similar incidence of adverse effects with respect to sex (data NR) | Low |
| 1 subgroup analysis of RCT[111] | 129 | 98 | Patients with active gastric ulcer treated with rabeprazole | Serious ^2,3^ | Incidence of adverse effects, changes in lab values and ECG | N/A | **Insignificant**  Similar incidence of adverse effects with respect to sex (data NR) | Low |
| **Statins - HMG-CoA Reductase Inhibitors** | | | | | | | | |
| 1 subgroup analysis of an RCT (women only)[36] | 4,855 | 3,390 | Patients with hypercholesteremia treated with lovastatin | Some^2^ | Patient-reported AEs, serious AEs, laboratory values | No | **Favors men**  No significant differences in laboratory values (ALT, CK) between men and women; however men had a lower overall incidence of AEs than women (p<0.01) and a lower incidence of clinical adverse effects that resulted in discontinuation of therapy: men 0.1%-0.9%; women 3.2%-3.7%, p<0.01. | Moderate |
| **Targeted Immune Modulators** | | | | | | | | |
| 1 Postmarketing surveillance[112] | 1,050 | 3,950 | Patients with active rheumatoid arthritis treated with infliximab | Serious^2,3^ | Adverse drug reactions | N/A | **None**  Similar rates of AEs between men and women, except that bacterial pneumonia occurred more frequently in men than in women (OR 1.94, 95% CI 1.29-2.93) | Very low |
| Alzheimer’s Drugs, Angiotensin Converting Enzyme Inhibitors, Angiotensin II Receptor Antagonists, Antiepileptic Drugs for Indications Other Than Epilepsy, Newer Antiemetics, Newer Antihistamines, Newer Antiplatelet Agents, Controller Medications for Asthma, Quick-relief Medications for Asthma, Pharmacological Treatments for ADHD, Atypical Antipsychotic Drugs, Beta Adrenergic Blockers, Beta2-Agonists, Calcium Channel Blockers, Constipation Drugs, Cyclo-oxygenase (COX)-2 Inhibitors and Non-steroidal Anti-inflammatory Drugs (NSAIDs), Newer Drugs for the Treatment of Diabetes Mellitus, Inhaled Corticosteroids, Newer Drugs for Insomnia, Pegylated Interferons for Chronic Hepatitis C Infection, Disease-modifying drugs for Multiple Sclerosis, Nasal Corticosteroids, Drugs for Neuropathic Pain, Long-Acting Opioid Analgesics, Oral Hypoglycemics, Skeletal Muscle Relaxants, Thiazolidinediones, Topical Cacineurin Inhibitors, Triptans | | | | | | | | |
| no evidence | | | | | | | | |

^1^ Publication bias is likely

^2^ Statistical limitations that could cause inferential error (e.g.no correction for multiple testing, no test of interaction, low power for subgroup analyses, not adjusted for confounders, no control group, post hoc data-analysis, no intention-to-treat population)

^3^ Limited to patient reported adverse effects

*High: Further research is very unlikely to change our confidence in the estimate of the effect; Moderate: Further research is likely to have an important impact on our confidence in the estimate of the effect and may change the estimate; Low: Further research is very likely to have an important impact on our confidence in the estimate of the effect and is likely to change the estimate; Very low: Any estimate of effect is very uncertain

AE: Adverse Effects; ALT: Alanine aminotransferase; CI: confidence interval; CK: Creatine kinase; ECG: Electrocardiogram; NNT: Number Needed to Treat; NR: Not Reported; OR: Odds; RR: Relative Risk; Ratio; Sex FX: Sex Effects Scale.
